# Earlier *In Vitro* Viral Production With SARS-CoV-2 Alpha Than With Beta, Gamma, B, or A.27 Variants

**DOI:** 10.3389/fcimb.2021.792202

**Published:** 2021-12-16

**Authors:** Samuel Lebourgeois, Houssem Redha Chenane, Nadhira Houhou-Fidouh, Reyene Menidjel, Valentine Marie Ferré, Gilles Collin, Nabil Benmalek, Romain Coppée, Lucile Larrouy, Yazdan Yazdanpanah, Jean-François Timsit, Charlotte Charpentier, Diane Descamps, Benoit Visseaux

**Affiliations:** ^1^ Université de Paris, Infection Antimicrobials Modelling Evolution (IAME), Institut National de la Santé et de la Recherche Médicale (INSERM), Paris, France; ^2^ Assistance Publique - Hôpitaux de Paris (AP-HP), University Hospital Bichat-Claude Bernard, Laboratoire de Virologie, Paris, France; ^3^ Assistance Publique - Hôpitaux de Paris (AP-HP), University Hospital Bichat-Claude Bernard, Maladies Infectieuses et Tropicales, Paris, France; ^4^ Assistance Publique - Hôpitaux de Paris (AP-HP), University Hospital Bichat-Claude Bernard, Réanimation Médicale et Infectieuses, Paris, France

**Keywords:** alpha, SARS-CoV-2, variants, replication cycle, infectious titres

## Abstract

Since its emergence in China at the end of 2019, SARS-CoV-2 has rapidly spread across the world to become a global public health emergency. Since then, the pandemic has evolved with the large worldwide emergence of new variants, such as the Alpha (B.1.1.7 variant), Beta (B.1.351 variant), and Gamma (P.1 variant), and some other under investigation such as the A.27 in France. Many studies are focusing on antibody neutralisation changes according to the spike mutations, but to date, little is known regarding their respective replication capacities. In this work, we demonstrate that the Alpha variant provides an earlier replication *in vitro*, on Vero E6 and A549 cells, than Beta, Gamma, A.27, and historical lineages. This earlier replication was associated with higher infectious titres in cell-culture supernatants, in line with the higher viral loads observed among Alpha-infected patients. Interestingly, Beta and Gamma variants presented similar kinetic and viral load than the other non-Alpha-tested variants.

## Introduction

In late 2019, severe acute respiratory syndrome coronavirus 2 (SARS-CoV-2), the etiological agent of coronavirus infectious disease 2019 (COVID-19), emerge worldwide. Following its rapid spread, SARS-CoV-2 was declared as pandemic by the World Health Organisation (WHO) on 11 March 2020 (Zhu et al., 2020). To date, COVID-19 has affected more than 200 countries with more than 250 million confirmed cases and more than 5 million deaths. SARS-CoV-2 infects the upper and lower respiratory tract, causing mild to severe respiratory syndromes (Harrison et al., 2020).

Since the beginning of these pandemic, several variants of SARS-CoV-2 have emerged. The first successful emergence was observed in March to April 2020 with the spread of the D614G mutation (Hodcroft et al., 2021). This mutation has been associated to higher viral loads and a better adhesion to the angiotensin-converting enzyme 2 (ACE2) cellular receptor (Korber et al., 2020). Since the late 2020, several new variants of concerns were identified. The Alpha variant, also known as the 20I/501Y.V1 or B.1.1.7 variant, has been firstly detected in London in December 2020 and rapidly spreading across Europe and worldwide. This variant is associated to higher viral loads and higher number of deaths (Challen et al., 2021 Davies et al., 2021). It presents, over all its genome, a total of 14 amino-acid substitutions and 3 deletions including several mutations in the S-glycoprotein, mainly the Δ69/70 and Δ144 deletions and the N501Y. Outside the S-glycoprotein, the main characteristic mutations are relating to ORF1 (T1001I, A1708D, I2230T, and Δ3665-3677), ORF8 (Q27stop, R25I, K68stop, and Y73C), and nucleocapsid (D3L, R203K, G204R/P, and S235F) (Duerr et al., 2021). The Beta, also known as the B.1.351, 20H/501Y.V2 or South-African variant, has been firstly detected in South-Africa in December 2020 and has also already spread worldwide. This Beta variant was phylogenetically distinct from the three main lineages (B.1.1.54, B.1.1.56, and C.1) circulating widely in South Africa during the first epidemic wave (Tegally et al., 2021a). The Beta has shown two mutations on the S-glycoprotein, the N501Y, also characterised in the Alpha variant, and the E484K mutation actually known to confer the antibody resistance against SARS-CoV-2 (Tegally et al., 2021b). Outside the S-glycoprotein, two other mutations relating to ORF 1 (K1655N) and nucleocapsid (T205I) on the viral genome are mainly described (Tegally et al., 2020; Garcia-Beltran et al., 2021). Similarly, the Gamma variant, also known as the P.1, 20J/501Y.V3 or Brazilian variant, shows both N501Y and E484k mutations with the addition of the K417T. The Gamma variant is responsible of a large new outbreak in Brazil, causing high mortality, and is currently spreading in Americas and Europe (Buss et al., 2021). Outside the S-glycoprotein, the main described mutations are relating to ORF1 (S1188L, K1798G, Δ3675-3677, and E5666D), ORF8 (E92K and 28269–28273 insertion), and nucleocapsid (P80R, R203K, G204R/P) (Sun et al., 2021). Another variant of interest, the A.27 or 19B/501Y, has also been recently detected and is slowly spreading in France. It is characterised into the S-glycoprotein by the absence of the D614G mutation but the presence of L452R and N501Y mutations that could improve viral transmission. Outside the S-glycoprotein, several other mutations could be identified in the N gene (S202N), ORF1a (P286L, D2980G, P1000L), ORF3a (V50A), and ORF8(L84S) (Fourati et al., 2021).

If the urgency of specific immunoglobulins and vaccine development has prompted the international community to look deeply for serum neutralisation studies and impact of new variants (Chen et al., 2020; Weisblum et al., 2020), there is still too little data on their infectivity and replication cycle. Such data are indeed of importance to explain some of their pathogenic aspects such as higher viral load or mortality (Challen et al., 2021).

In this work, we sought to study the replicative capacity of a historical B strain along with Alpha, Beta, Gamma, and A.27 variants currently circulating in France and worldwide. Our experiments were conducted in the widely spread Vero E6 cell line model and confirmed in human A549 lung cell line expressing the ACE-2 receptor and TMPRSS2 coreceptor. We observed several differences in replication and infectious viral particle production rates, especially with the Alpha variant, that should play a part in its higher viral loads and death rates.

## Materials and Methods

### Cell Lines and Viral Lineages

The Vero E6 cell line was obtained from the American Type Culture Collection (ATCC, reference R CRL-1586) (LGC standards SARL, Illkirch, France) and cultured in Dulbecco’s modified Eagle’s medium (DMEM, Gibco™) supplemented with 10% of heat-inactivated foetal bovine serum (FBS, Gibco™) (Thermo Fisher Scientific, Waltham, MA, 209 USA). The A549 enriched with human ACE2 and TMPRSS-2 surface proteins was obtained from *In vivo*Gen^©^ and cultured in the same medium than Vero E6 cell line, added with puromycin and hygromycin B as recommended. Both cell lines were incubated at 37°C in a humidified atmosphere with 5% of CO_2_. The viral strains of human SARS-CoV-2 variant were obtained from a positive nasopharyngeal PCR sample. The viruses have been treated in biosafety level-3 laboratory (BSL-3). The SARS-CoV-2 primo-culture stocks used as B (*n* = 1 viral strain) (EPI_ISL_4537783), Alpha (*n* = 2) (EPI_ISL_4536454 and EPI_ISL_4536996), Beta (*n* = 2) (EPI_ISL_4537125 and EPI_ISL_4537284), Gamma (*n* = 1) (EPI_ISL_4536760), and A.27 (*n* = 1) (EPI_ISL_4537460) was produced in Vero E6 cells. The supernatant were quantified with viral RNA levels and titrated by lysis plaque assay (Gordon et al., 2020), aliquoted, and stored at −80°C.

### Kinetic and Viral Infection Assays

Vero E6 and A549 cells were seeded onto 12-well plates at a density of 100,000 cells per well for Vero E6 cells or 200,000 cells per well for A549. For all virus strains, 18 h postseeding, cells were infected with multiplicity of infection (MOI) titres of 0.01. Briefly, cells were washed once with a serum-free medium and infected with 500 µl of a SARS-CoV-2 serum-free medium viral suspension. After virus adsorption for 1 h at 37°C, the viral inoculum was removed and the cells were washed with a FBS-free medium. A total of 1 ml of DMEM supplemented with 2% FBS was then added onto the infected cells. For each tested condition, three corresponding wells were used to collect cells and supernatants on a daily basis. After centrifugation, cells were washed three times with PBS. Cells and supernatants were immediately tested for viral SARS-CoV-2 RNA and albumin cell DNA PCR, allowing quantifying viral copy genome number per million cells. The remaining samples were stored at −80°C before testing by N antigen titration and infectious titre evaluation by lysis plaque assay as depicted below. Each assay was performed with three replicates conducted independently for each tested viral strain.

### Nucleic Acid Extraction and Quantitative PCR

Both cells and supernatants were extracted from 100 µl of each supernatant or resuspended cells sample with the Total NA Isolation kit - Large Volume assay on a MagNA Pure LC 2.0 analyser (Roche, Basel, Switzerland). All nucleic acids were eluted in a 50-µl elution buffer. A quantitative PCR of the albumin gene with a standard human DNA (0.2 µg/µl) dilution, for quantifying cellular DNA, was performed as previously described (Desire et al., 2001). The SARS-CoV-2 RNA was quantified from 10 µl of extracted samples with the RealStar™ SARS-CoV-2 RT-PCR Kit 1.0 assay (Altona Diagnostics GmbH, Hamburg, Germany) (Visseaux et al., 2020). The viral quantification was performed using a standardised RNA transcript control obtained from the European Virus Archive Program and targeting E gene as previously described (Visseaux et al., 2020). Moreover, an internal control was used in all PCR assay to check the absence of PCR inhibitors.

### Viral Titration

SARS-CoV-2 was titrated by a lysis-plaque assay as previously described (Gordon et al., 2020). Briefly, Vero E6 cells were seeded onto a 12-well plate at a density of 100,000 in DMEM with 10% FBS. The next day, cells were infected by 10 to 10 serial viral dilutions with the same infection protocol than for our viral infection assays. After the viral adsorption period of 1 h at 37°C, 500 µl of an agarose medium mix was added. After 3-day incubation at 37°C with 5% of CO_2_, the supernatant was removed and cells were fixed with 1 ml to 6% of formalin solution for 30 min. The formalin solution was then removed, and cells were coloured with a 10% crystal violet solution for 15 min. All wells were then washed with distilled water and dried on bench-coat paper.

### N-Antigen Level Assessment

N-antigenemia levels were determined with a being marketed CE-IVD ELISA microplate assay, COV-Quanto^®^ (AAZ, Boulogne-Billancourt, France), according to manufacturer recommendations (Le Hingrat et al., 2020). Briefly, in each well of 96-well microplates, coated with anti-SARS-CoV-2 N-antibodies, 50 µl of a solution containing biotinylated anti-SARS-CoV-2 N antibodies and 50 µl of cells or supernatants were added. After incubation at 37°C for 60 min, 100 µl of a solution containing HRP-conjugated streptavidin were added, followed by a 30-min incubation at 37°C. After being washed, 50 µl of a solution containing the peroxide substrate and 50 µl of a second substrate (3,3′,5,5′-tetramethylbenzidine (TMB)) were then added. After 15 min at 37°C, the colorimetric reaction was stopped by adding 50 µl of H_2_SO_4_. Absorbance values were measured at 450 nm, with a reference set at 630 nm. Standards, made of recombinant N antigens, were added to each microplate, as recommended by the manufacturer, to allow N-antigenemia-level determination.

### Statistical Analysis

For each time point, potential differences were tested across all lineage groups using the Kruskal-Wallis test. Differences between two lineages were then tested using a Mann-Whitney *U* test. All statistics were calculated using R 4.1.0.

### Ethical Consideration

According to current French ethical laws and regulations, written informed consents are not required for viral strain characterisation.

## Results

**Figure 1:**
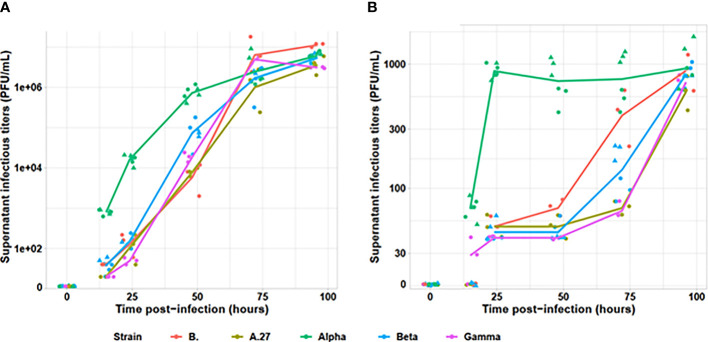
Evaluation of infectious viral particle production kinetics for tested viral variants. The infectious titers, determined by plaque assay on **(A)** Vero E6 and **(B)** A549 cell lines, are indicated on a logarithmic scale. The Alpha variant is indicated in green, the Beta in blue, the Gamma in purple, the A.27 in khaki and the B strain in red. For Alpha and Beta, two strains were tested and are identified by the dot triangle or circle shapes. Each strain was tested by two independent replicates.

The Alpha variant presented an earlier production of infectious viral particles than the other tested viral variants on both Vero E6 and A549 cell lines (cf. **Figure 1**). Indeed, as early as 15 h after infection, the infectious particle production was statistically different across strains (*p* = 0.005). All non-Alpha strains presented similar viral particles amounting up to 40 PFU/ml (*p* = 0.19), statistically lower than the Alpha variant presenting at 600 to 900 PFU/ml (*p* = 0.005). At 24 h, all non-Alpha strains produced similar detectable viral particle amount comprising between 2 and 220 PFU/ml (*p* = 0.24). However, the two Alpha strains produced from 18,000 to 21,000 PFU/ml across replicates, statistically higher than non-Alpha variants (*p* = 0.02). Similar pattern was also observed at 24 and 48 h, with similar levels for all non-Alpha strains (*p* = 0.24 and *p* = 0.19, respectively) and statistically higher levels for Alpha strains (*p* < 0.001 and *p* = 0.009, respectively). We observed similar infectious titres for all strains at 72 h, plateauing around 10^7^ PFU/ml. Those observations were confirmed on A549 human pulmonary cells, despite lower levels of infectious viral particle production, as Alpha strain was able to produce significant amount of infectious viral particles at 15 h, statistically higher than the very low amount of non-Alpha strains (*p* = 0.001). Similar levels of infectious viral particles were only observed for all strains at 96 h.

**Figure 2:**
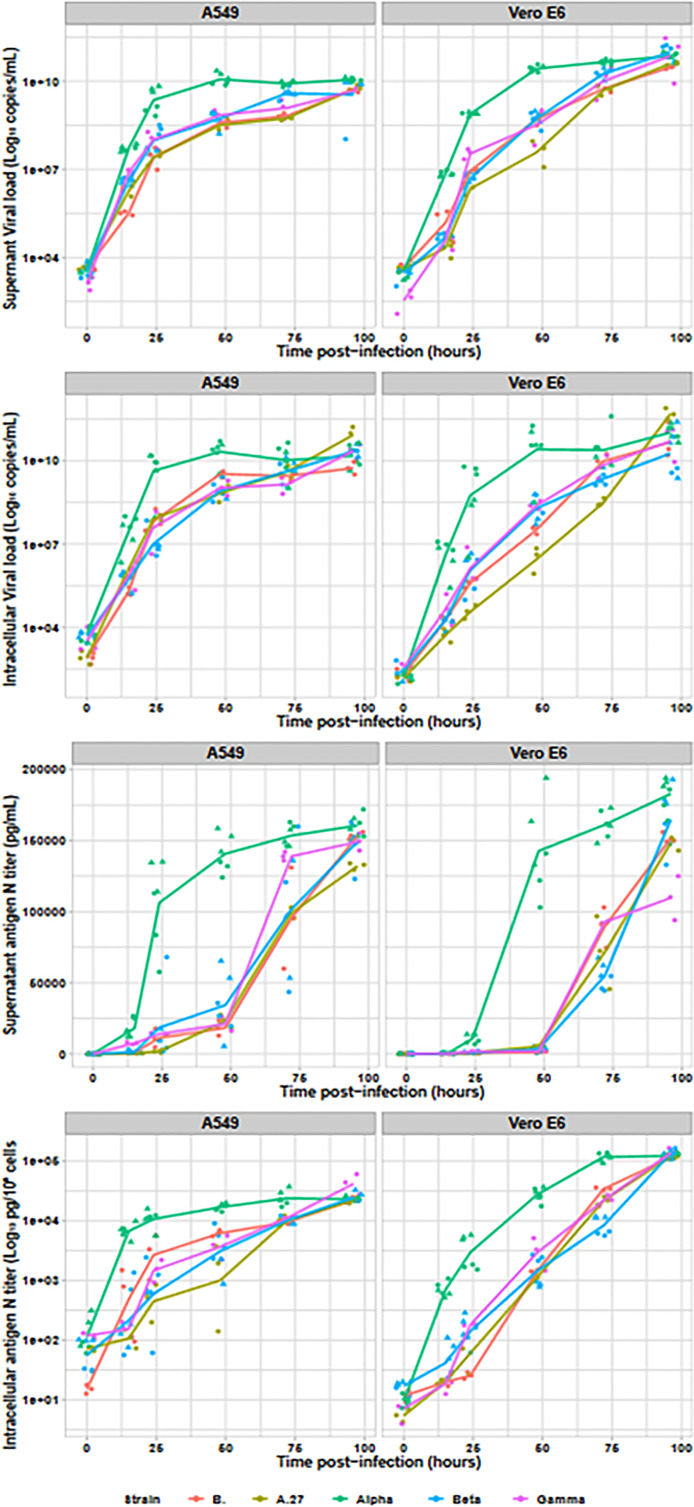
Viral replication assessment in culture supernatant and intracellular fraction. The Alpha variant is indicated in green (n=6), the Beta in blue (n=6), the Gamma in purple (n=3), the A27 in khaki (n=3) and the B. strain in red (n=3). For Alpha and Beta, two strains were tested and are identified by the dot triangle or circle shapes. With the exception of the N antigen in culture supernatant, all data were plotted on logarithmic scales.

Assessment of SARS-CoV-2 viral production was also performed by RNA and antigen N intracellular and extracellular productions (cf. **Figure 2**). RNA in culture supernatant provided earlier kinetics than supernatant infectious titres or intracellular RNA. Moreover, the Alpha variant produced around 10 times higher RNA loads than the other strains, both in culture supernatant (Vero E6: *p* < 0.001; A549: *p* < 0.001) and intracellular fraction (Vero E6: *p* < 0.001; A549 p < 0.001). All non-Alpha viral strains produced similar amounts of viral RNA at 15 h in both supernatant (Vero E6: *p* = 0.26; A549: *p* = 0.07) and intracellular RNA (Vero E6: *p* = 0.15; A549: *p* = 0.06). Similar pattern was also observed at 24 and 48 h, with similar levels for all non-Alpha strains (*p* = 0.08 and *p* = 0.07, respectively), despite slightly lower levels for the A.27 strain, and statistically higher levels for Alpha strains (*p* < 0.001 and *p* < 0.001, respectively) on Vero E6 cells. The same profile has been confirmed on A549 cell line. Finally, all RNA levels reached similar plateaus for all tested variants after 72 h of infection. Production of N antigen in the culture supernatant provided similar kinetics to infectious viral particle titres with statistically earlier Alpha viral N-antigen production since 15 h after infection up to a final plateau at 48 h postinfection while the other strains reached similar plateaus but only at 72 h postinfection. Intracellular N-antigen levels detected at 15 h postinfection was also similar for all non-Alpha strains on both cell lines (Vero E6: *p* = 0.11; A549: *p* = 0.57), but statistically lower than for the two Alpha strains with levels between 5.12 × 10^2^ and 9.85 × 10^3^ pg/10^5^ cells (Vero E6: *p* < 0.001; A549: *p* < 0.001). This pattern was also observed in supernatant N antigen at 24 and 48 h, as well as for intracellular N-antigen levels.

## Discussion

The emergence of new SARS-CoV-2 variants, with several data suggesting higher viral loads and/or better resistance to seroneutralisation, is forcing the scientific community to quickly react and provide new data for assessing and understanding the new threats. In the current work, we investigated the viral replication kinetics and production of viral particles of four variants, Alpha, Beta, Gamma, and A.27, in parallel to the historical strain B. We highlight here, a shorter replication cycle and quicker production of infectious viral particle with the Alpha (i.e., the UK variant) than with B strain (a historical variant), Beta (i.e., the South-African variant), Gamma (i.e., the Brazilian variant), or A.27 variant, a recent variant under investigation observed in France.

Many efforts are focusing on the response of variants to immunoglobulin and vaccines (Planas et al., 2021; Zhou et al., 2021), a cornerstone question for public health policies. However, the potential differences among variants on the global viral fitness have not been characterised to date. This is of importance, especially as the Alpha variant provides higher viral loads and higher death rates than the historical strains (Challen et al., 2021; Davies et al., 2021) and as we are lacking such data on the other newly emerged variants. For the Alpha variant, the higher viral loads seem associated with earlier consultation since patients are consulting one day earlier since symptoms onset than with historical variants (Challen et al., 2021; Davies et al., 2021). It is unclear how these two facts are related.

Our results confirm those epidemiological observations. The almost 10 times higher *in vitro* production of viral RNA observed with the Alpha strain between 15 and 48 h postinfection is in line with the higher viral loads observed in the UK (Davies et al., 2021; Challen et al., 2021). We also observed earlier production of infectious viral particles with the Alpha strain, 1 day before the other variants and even detectable since 15 h postinfection. This shorter *in vitro* replication cycle is in line with the earlier consultation observed in the UK (Challen et al., 2021; Davies et al., 2021).

Our results have been confirmed on two cellular models, the reference Vero E6 model and the A549 human pulmonary immortalised cell line, as well as with different markers including viral RNA or N-antigen production in the culture supernatant and within the cells. Interestingly, in the culture supernatant, the results observed with the N antigen were much closer to the infectious titres than the viral RNA detection. This could be explained by high levels of genomic and mRNA since the earliest stage of cell infection which can be released by the cells but do not reflect the presence of viable viral particles. When studying the cellular layers, the N-antigen titres were remarkably similar to intracellular RNA measurements, suggesting that they adequately reflect viral accumulation within the infected cells. Thus, N-antigen measurements appear to provide a quick, easy, and highly informative tool for SARS-CoV-2 cellular culture assessment. The detectable levels of intracellular N-antigen and RNA immediately after cellular infection should also reflect the viral entry during the infection steps. Thus, the higher levels observed with the A549 cells than Vero E6 are in line with the presence of the TMPRSS2 coreceptor expressed by those former cells.

To date, only little preliminary data are available on replicative advantage of the recent variants of concerns. A study in a mice model depicts a lower lung viral load for B.1 and Alpha than Beta and Gamma (Montagutelli et al., 2021). In another preprint study, conducted in hamster model, low Alpha lung infectious particle titres were also identified but, on the contrary, with higher Alpha nasal infectious titres than several B.1 strains (HK-15, GH 405, and HK-95) (Mok et al., 2021). Using Vero and primary human airway epithelial cells, Brown et al. did not evidence a replicative advantage of Alpha cells over some other variants but did not tested historical B strains nor Beta or Gamma (Brown et al., 2021). All these results will need further confirmation. Our study provides complementary data on two reference cell lines, Vero E6 and human airway A549-ACE2-TMPRSS2 cell lines, using standardised viral inoculum and several complementary replication measurement methods. Our observation, of quicker high viral load and infectious titres for Alpha, are also in line with the higher viral loads also observed in France (Teyssou et al., 2021) and culture positivity rates from clinical nasopharyngeal samples evidenced in a recent larger German study (Jones et al., 2021). In our work, we also tested the three more widely spread variants of concerns that do not show quicker or stronger replication capabilities. This reassuring observation is in line with a first study including clinical viral load data for Beta, presenting lower viral loads than for the Alpha variant (Teyssou et al., 2021), but will need to be confirmed by other larger clinical studies.

The current study presents several limitations. Despite the use of two different cell lines, including one immortalised human respiratory cell line, those observations should be confirmed on primary respiratory cells. As the viral variants studied present numerous differential mutations over their whole genomes. If the international research effort is mainly focusing on S-glycoprotein mutations, for predicting viral adhesion to cellular receptors and viral escape to neutralising antibodies, the mutations found in all the other genes are expected to play major roles in viral fitness differences. Further studies will be needed to correlate the phenotypic differences observed with any mutation or combination of those mutations. In our work, highlighting the viral fitness of Alpha variant, we found only one additional mutation compared with the archetypal Alpha strains for the first tested strain: ORF1b P314L, which is not described in the literature to our knowledge (GISAID: EPI_ISL_4536454). The second tested Alpha strain depicts the same mutation, along with ORF1a E1363G, ORF1b S2027L, and ORF3a W131C (GISAID: EPI_ISL_4536996). Due to the absence of kinetic difference between our two strains, we do not believe that they play any role in the shorter Alpha replication rate. The individual role of all mutations observed between the tested variants, and their associations, still have to be described by further studies.

In conclusion, we highlight in this work a shorter replication cycle and a quicker production of infectious viral particle with the Alpha than with several other variants currently circulating or emerging in France and worldwide. This is expected to play a role and explain a part of the higher viral loads, higher mortality rates, and earlier consultations observed in the UK and Germany with the Alpha. The comparable replication cycles observed for all the other variants tested in this work is also reassuring regarding their fitness and virulence, but will need to be confirmed by large cohort studies as done in the UK.

## Data Availability Statement

The original contributions presented in the study are included in the article/supplementary material. Further inquiries can be directed to the corresponding author.

## Author Contributions

SL, HC, and BV contributed to conception and design of the study. RM and SL organized the database. SL performed the statistical analysis. SL and BV wrote the first draft of the manuscript. SL, BV, and HC wrote sections of the manuscript. All authors contributed to manuscript revision and read and approved the submitted version. All authors contributed to the article and approved the submitted version.

## Funding

This study has been funded in part by AC43 of the French Agence Nationale de Recherche sur le SIDA et les hépatites virales (ANRS).

## Conflict of Interest

The authors declare that the research was conducted in the absence of any commercial or financial relationships that could be construed as a potential conflict of interest.

## Publisher’s Note

All claims expressed in this article are solely those of the authors and do not necessarily represent those of their affiliated organizations, or those of the publisher, the editors and the reviewers. Any product that may be evaluated in this article, or claim that may be made by its manufacturer, is not guaranteed or endorsed by the publisher.

## References

[B1] Brown J. C. Goldhill D. H. Zhou J. Peacock T. P. Frise R. Goonawardane N.. (2021). Increased Transmission of SARS-CoV-2 Lineage B.1.1.7 (VOC 2020212/01) Is not Accounted for by a Replicative Advantage in Primary Airway Cells or Antibody Escape. bioRxiv, 2021.02.24.432576. doi: 10.1101/2021.02.24.432576

[B2] Buss L. F. Prete C. A. Abrahim C. M. M. Mendrone A. Salomon T. Almeida-Neto C.. (2021). Three-Quarters Attack Rate of SARS-CoV-2 in the Brazilian Amazon During a Largely Unmitigated Epidemic. Science 371, 288 –2292. doi: 10.1126/science.abe9728 33293339 PMC7857406

[B3] Challen R. Brooks-Pollock E. Read J. M. Dyson L. Tsaneva-Atanasova K. Danon L. (2021). Risk of Mortality in Patients Infected with SARS-CoV-2 Variant of Concern 202012/1: Matched Cohort Study. BMJ 372, n579. doi: 10.1136/bmj.n579 33687922 PMC7941603

[B4] Chen X. Li R. Pan Z. Qian C. Yang Y. You R.. (2020). Human Monoclonal Antibodies Block the Binding of SARS-CoV-2 spike Protein to Angiotensin Converting Enzyme 2 Receptor. Cell. Mol. Immunol. 1,–3. doi: 10.1038/s41423-020-0426-7 PMC7167496 32313207

[B5] Davies N. G. Jarvis C. I. CMMID COVID-19 Working Group Edmunds W. J. Jewell N. P. Diaz-Ordaz K.. (2021). Increased Mortality in Community-Tested Cases of SARS-CoV-2 Lineage B.1.1.7. Nature. doi: 10.1038/s41586-021-03426-1 PMC9170116 33723411

[B6] Desire N. Dehee A. Schneider V. Jacomet C. Goujon C. Girard P.-M.. (2001). Quantification of Human Immunodeficiency Virus Type 1 Proviral Load by a TaqMan Real-Time PCR Assay. J. Clin. Microbiol. 39, 1303 –11310. doi: 10.1128/JCM.39.4.1303-1310.2001 11283046 PMC87929

[B7] Duerr R. Dimartino D. Marier C. Zappile P. Wang G. Lighter J.. (2021). Dominance of Alpha and Lota Variants in SARS-CoV-2 Vaccine Breakthrough Infections in New York City. J. Clin. Invest. 131:152702. doi: 10.1172/JCI152702 34375308 PMC8439605

[B8] Fourati S. Decousser J.-W. Khouider S. N'Debi M. Demontant V. Trawinski E.. Early Release - Novel SARS-CoV-2 Variant Derived from Clade 19B, France - Volume 27, Number 5 –May 2021 - Emerging Infectious Diseases journal - CDC. doi: 10.3201/eid2705.210324 PMC8084519 33900195

[B9] Garcia-Beltran W. F. Lam E. C. St. Denis K. Nitido A. D. Garcia Z. H. Hauser B. M.. (2021). Multiple SARS-CoV-2 Variants Escape Neutralization by Vaccine-induced Humoral Immunity. Cell 184, 2372–2383.e9. doi: 10.1016/j.cell.2021.03.013 33743213 PMC7953441

[B10] Gordon D. E. Jang G. M. Bouhaddou M. Xu J. Obernie2r K. White K. M.. (2020). A SARS-CoV-2 Protein Interaction Map Reveals Targets for Drug Repurposing. Nature 583, 459 –4468. doi: 10.1038/s41586-020-2286-9 32353859 PMC7431030

[B11] Harrison A. G. Lin T. Wang P. (2020). Mechanisms of SARS-CoV-2 Transmission and Pathogenesis. Trends Immunol. 41, 1100 –11115. doi: 10.1016/j.it.2020.10.004 33132005 PMC7556779

[B12] Hodcroft E. B. Zuber M. Nadeau S. Vaughan T. G. Crawford K. H. D. Althaus C. L.. (2021). Emergence and Spread of a SARS-CoV-2 Variant Through Europe in the Summer of 2020. MedRxiv Prepr. Serv. Health Sci., 2020.10.25.20219063. doi: 10.1101/2020.10.25.20219063

[B13] Jones T. C. Biele G. Mühlemann B. Veith T. Schneider J. Beheim-Schwarzbach J.. (2021). Estimating Infectiousness Throughout SARS-CoV-2 Infection Course Science. doi: 10.1126/science.abi5273 PMC9267347 34035154

[B14] Korber B. Fischer W. M. Gnanakaran S. Yoon H. Theiler J. Abfalterer W.. (2020). Tracking Changes in SARS-CoV-2 Spike: Evidence that D614G Increases Infectivity of the COVID-19 Virus. Cell 182, 812–827.e19. doi: 10.1016/j.cell.2020.06.043 32697968 PMC7332439

[B15] Le Hingrat Q. L. Visseaux B. Laouenan C. Tubiana S. Bouadma L. Yazdanpanah Y.. (2020). Detection of SARS-CoV-2 N-Antigen in Blood During Acute COVID-19 Provides a Sensitive New Marker and New Testing Alternatives. Clin. Microbiol. Infect. Off. Publ. Eur. Soc Clin. Microbiol. Infect. Dis. doi: 10.1016/j.cmi.2020.11.025 PMC7724284 33307227

[B16] Mok B. W.-Y. Liu H. Lau S.-Y. Deng S. Liu S. Tam R. C.-Y.. (2021). Low Dose Inocula of SARS-CoV-2 B.1.1.7 Variant Initiate More Robust Infections in the Upper Respiratory Tract of Hamsters than Earlier D614G Variants. bioRxiv, 2021.04.19.440414. doi: 10.1101/2021.04.19.440414

[B17] Montagutelli X. Prot M. Levillayer L. Salazar E. B. Jouvion G. Conquet L.. (2021). The B1.351 and P.1 Variants Extend SARS-CoV-2 Host Range to Mice. bioRxiv, 2021.03.18.436013. doi: 10.1101/2021.03.18.436013

[B18] Planas D. Bruel T. Grzelak L. Guivel-Benhassine F. Staropoli I. Porrot F.. (2021). Sensitivity of Infectious SARS-CoV-2 B.1.1.7 and B.1.351 Variants to Neutralizing Antibodies. Nat. Med. doi: 10.1038/s41591-021-01318-5 33772244

[B19] Sun S. Gu H. Cao L. Chen Q. Ye Q. Yang G.. (2021). Characterization and Structural Basis of a Lethal Mouse-Adapted SARS-CoV-2. Nat. Commun. 12, 5654. doi: 10.1038/s41467-021-25903-x 34580297 PMC8476561

[B20] Tegally H. Wilkinson E. Giovanetti M. Iranzadeh A. Fonseca V. Giandhari J.. (2020). Emergence and Rapid Spread of a New Severe Acute Respiratory Syndrome-Related Coronavirus 2 (SARS-CoV-2) Lineage With Multiple Spike Mutations in South Africa. doi: 10.1101/2020.12.21.20248640

[B21] Tegally H. Wilkinson E. Giovanetti M. Iranzadeh A. Fonseca V. Giandhari J.. (2021a). Detection of a SARS-CoV-2 Variant of Concern in South Africa. Nature. doi: 10.1038/s41586-021-03402-9 33690265

[B22] Tegally H. Wilkinson E. Lessells R. J. Giandhari J. Pillay S. Msomi N.. (2021b). Sixteen Novel Lineages of SARS-CoV-2 in South Africa. Nat. Med. 27, 440 –4446. doi: 10.1038/s41591-021-01255-3 33531709

[B23] Teyssou E. Soulie C. Visseaux B. Lambert-Niclot S. Ferre V. Marot S.. (2021). The 501Y.V2 SARS-CoV-2 Variant has an Intermediate Viral Load Between the 501Y.V1 and the Historical Variants in Nasopharyngeal Samples from Newly Diagnosed COVID-19 Patients. J. Infect. doi: 10.1016/j.jinf.2021.04.023 PMC8080495 33932451

[B24] Visseaux B. Le Hingrat Q. Collin G. Ferré V. Storto A. Ichou H.. (2020). Evaluation of the RealStar® SARS-CoV-2 RT-PCR kit RUO Performances and Limit of Detection. J. Clin. Virol. Off. Publ. Pan Am. Soc Clin. Virol. 129:104520. doi: 10.1016/j.jcv.2020.104520 PMC7323686 32652476

[B25] Weisblum Y. Schmidt F. Zhang F. DaSilva J. Poston D. Lorenzi J. C.. (2020). Escape from Neutralizing Antibodies by SARS-CoV-2 Spike Protein Variants. eLife 9. doi: 10.7554/eLife.61312 PMC7723407 33112236

[B26] Zhou D. Dejnirattisai W. Supasa P. Liu C. Mentzer A. J. Ginn H. M.. (2021). Evidence of Escape of SARS-CoV-2 Variant B.1.351 From Natural and Vaccine-Induced Sera. Cell. doi: 10.1016/j.cell.2021.02.037 PMC7901269 33730597

[B27] Zhu N. Zhang D. Wang W. Li X. Yang B. Song J.. (2020). A Novel Coronavirus From Patients With Pneumonia in Chin. N. Engl. J. Med. 382, 727–733. doi: 10.1056/NEJMoa2001017 31978945 PMC7092803

